# Sixty Years (1957–2017) of Research on Toxoplasmosis in China—An Overview

**DOI:** 10.3389/fmicb.2017.01825

**Published:** 2017-09-25

**Authors:** Ming Pan, Congcong Lyu, Junlong Zhao, Bang Shen

**Affiliations:** ^1^State Key Laboratory of Agricultural Microbiology, College of Veterinary Medicine, Huazhong Agricultural University Wuhan, China; ^2^Key Laboratory of Preventive Medicine in Hubei Province Wuhan, China; ^3^Hubei Cooperative Innovation Center for Sustainable Pig Production Wuhan, China

**Keywords:** *Toxoplasma gondii*, epidemiology, China, vaccine, genotype

## Abstract

*Toxoplasma gondii* is a ubiquitous zoonotic pathogen belonging to apicomplexan parasites. Infection in humans and animals may cause abortion and other severe symptoms under certain circumstances, leading to great economical losses and public health problems. *T. gondii* was first discovered in China in 1955 and the corresponding work was published in 1957. Since then, a lot of work has been done on this parasite and the diseases it causes. This review summarizes the major progress made by Chinese scientists over the last 60 years, and gives our perspectives on what should be done in the near future. A wide variety of diagnostic approaches were designed, including the ones to detect *T. gondii* specific antibodies in host sera, and *T. gondii* specific antigens or DNA in tissue and environmental samples. Further work will be needed to translate some of the laboratory assays into reliable products for clinic uses. Epidemiological studies were extensively done in China and the sero-prevalence in humans increased over the years, but is still below the world average, likely due to the unique eating and cooking habits. Infection rates were shown to be fairly high in meat producing animals such as, pigs, sheep, and chickens, as well as in the definitive host cats. Numerous subunit vaccines in the form of recombinant proteins or DNA vaccines were developed, but none of them is satisfactory in the current form. Live attenuated parasites using genetically modified strains may be a better option for vaccine design. The strains isolated from China are dominated by the ToxoDB #9 genotype, but it likely contains multiple subtypes since different ToxoDB #9 strains exhibited phenotypic differences. Further studies are needed to understand the general biology, as well as the unique features of strains prevalent in China.

## Introduction

*Toxoplasma gondii* is an obligate intracellular parasitic protozoan widely distributed around the world. One-third of the world's population was estimated to be infected by this parasite (Dubey, 2010). In addition to humans, it can also infect more than 200 species of animals and causes toxoplasmosis in them (Gao, 2014). As an opportunistic pathogen, *T. gondii* infection rarely causes severe symptoms in healthy humans and most other hosts. The main risks are congenital infections during pregnancy, which may cause abortion, stillbirth, developmental defects, and other serious diseases to the fetus. Congenital infections and subsequent complications are common in humans and farm animals such as, pigs and sheep, leading to great economical losses and social problems (Dubey, 2009).

One important reason for the wide spread of *T. gondii* is its complex life cycle, which consists a sexual phase and an asexual phase. Sexual reproduction occurs in definitive hosts, the felids. After infection, they shed oocysts in feces to contaminate water and environment and pass the infection to other hosts if oocysts are ingested. In intermediate hosts, the parasites propagate asexually and they can be transmitted between intermediate hosts through predation. A large portion of human toxoplasmosis cases were thought to be derived from consumption of undercooked meat that was infected (Montoya and Liesenfeld, 2004).

**Figure 1:**
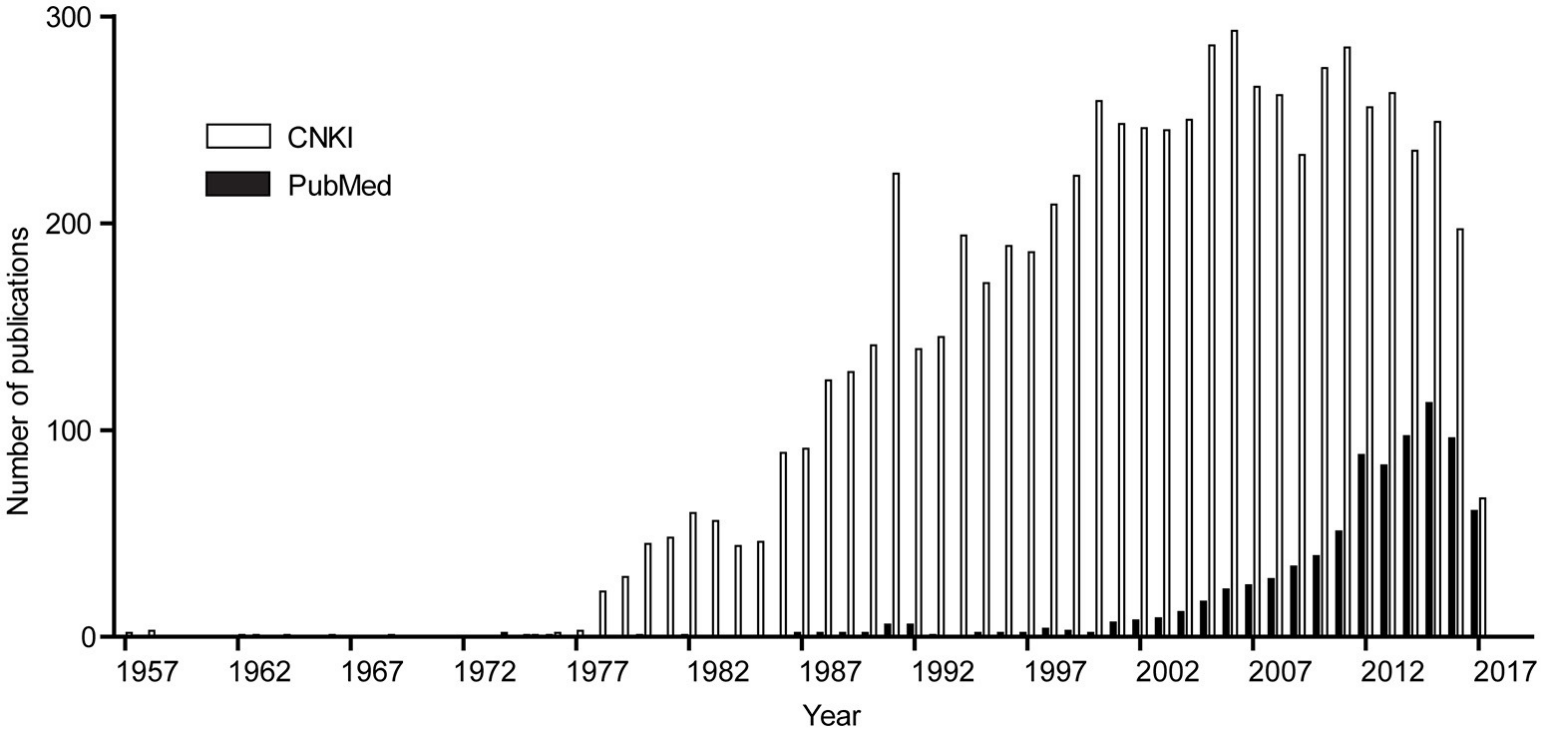
Number of papers published by scientists in China each year since 1957. The key word “*Toxoplasma*” and affiliation “China” were used to search the PubMed database (https://www.ncbi.nlm.nih.gov/pubmed/) for papers published by scientists in China in international journals. The keywords “*Toxoplasma*” or “toxoplasmosis” or the Chinese equivalent of the two were used to search the Chinese library CNKI (http://www.cnki.net/) to estimate the papers in Chinese journals. Subsequently the numbers of papers from the two searches were reported by year.

China has a long history of *T. gondii* research, which dates back to 1955 when Yu et al first isolated *T. gondii* from cats and rabbits in Fujian Province. This work was published in the Chinese journal *Acta Microbiologica Sinica* in 1957 (Yu et al., 1957). After this discovery, more and more work has been done to understand the epidemiology and biology of this parasite in China. When the key words “*Toxoplasma”* (or “toxoplasmosis”) and “China” were used to search the PubMed database (English articles) and the China National Knowledge Infrastructure (CNKI) database (Chinese articles), we estimated the number of publications on *Toxoplasma* or toxoplasmosis in each year from the Chinese research community and the results are summarized in Figure 1. There were only sporadic reports published before 1978, but since then the research activities became more active (Figure 1). There are at least two reasons for that: first, during late 1970s, many pigs in Shanghai and the nearby area were reported to have a fatal syndrome called “high fever with unknown reason,” and later it was shown to be caused by *T. gondii* infection (Ren and Bao, 1982), which promoted studies on this parasite; second, the implementation of the *Economic Reform and Open up* policies in 1978 in general boosted scientific researches. In this review, we collected all the studies published in English and Chinese journals and summarized the work that have been done on *Toxoplasma* and toxoplasmosis in China over the last 60 years. A perspective was also outlined for areas of research in the future.

## Development of diagnostic approaches

Because of the lack of unique symptoms or traits, diagnosis of toxoplasmosis is challenging. This is particularly true for human infections during pregnancy, since the risks of which depend on when exactly the infection is acquired. Most European and North American countries have reference laboratories for the diagnosis and risk assessments. This concept has been introduced into China, but it never really works the way as it should, since there is no bona fide reference labs operating at the national level. Nonetheless, over the last 40 years, Chinese scientists have done a lot of work to develop diagnostic strategies to detect *T. gondii* infections. They can be mainly grouped into three types: serological tests detecting *T. gondii* specific antibodies post infection, immunological methods detecting *T. gondii* specific antigen, and nucleic acids based pathogen detection. Representative methods for each type were listed in Table 1.

**Table 1:** Diagnostic methods developed by scientists in China.

**Detecting**	**Methods**	**Sensitivity and specificity (host)**	**References**
*T. gondii* specific Antibodies	**Indirect-ELISA, using:** Truncated recombinant SAG1 Multi-epitope peptide derived from SAG1, SAG2 and SAG3 GRA1, GRA7 MIC3	93.9% (31/33) positive concordance compared with Western blot (100%) (human) The total concordance was 93.2 and 95.7% for the detection of IgG and IgM antibodies, respectively, compared with commercial ELISA tests (human) Highly sensitive (93.2%) and specific (94.0%) (dog) Higher positive detections than MAT in pigs and goats	Wu et al., 2009 Dai et al., 2013 Wang Z. et al., 2014 Zhang et al., 2010
	**Dynamic flow immunochromatographic Test (DFICT)** using SAG1 and SAG2	The lowest detectable limit was 1:320 dilution of positive serum (dog and cat)	Jiang W. et al., 2015
	**Latex agglutination tests (LAT)** using MIC3	After 8 days infection, *T. gondii* specific antibodies could be detected (pig)	Jiang et al., 2008
	**Indirect hemagglutination assays (IHA)** using soluble parasite antigen sensitized sheep red blood cells	*T. gondii* specific antibodies could be detected on the 7th days after initial infection (rabbit)	Chen et al., 1980
	**Piezoelectric immunoagglutination assay** using total antigen of the RH strain	The detectable limit was 1:5,500 dilution of positive serum (rabbit)	Wang et al., 2004
	**Fast dipstick dye immunoassay (DDIA)** using total soluble antigen of the RH strain	Sensitivity and specificity of IgG were 100 and 96%, respectively, while sensitivity and specificity of IgM were 100 and 94%, respectively (human)	Jin et al., 2005
*T. gondii* Antige**n**s	**Modified rapid sandwich ELISA** using rabbit polyclonal antibodies against soluble *T. gondii* antigens	Detected circulating antigens at the concentration of 31.2 ng/mL, and no cross-reaction with other protozoa (human and rabbit)	Chen R. et al., 2008
	**Immunochromatographic strip** using sheep antisera against excretory/secretory antigens of *T. gondii* circulating antigens	Circulating antigens could be detected from day 2 to day 14 (or even longer) after parasite infection (pig, goat, and sheep)	Wang et al., 2011
*T. gondii* DNA	**LAMP** targeting 529 bp repeat sequence, SAG1 gene or B1 gene	Superior sensitivity than the conventional PCR, can detected *T. gondii* at the concentration of 2–3 parasites/mL, highly specific (pig, sheep, mouse, rabbit)	Yang et al., 2008; Lin et al., 2012; Lai et al., 2014
	**RT-LAMP** targeting 18s RNA gene	Showed higher sensitivity than RT-PCR with the detection limit of 1 tachyzoite in 1 g pork (pig)	Qu et al., 2013b
	**RT-PCR** targeting 529 bp repeat sequence	The sensitivity was as higher (1 fg DNA), compared to conventional PCR (100 fg) (pig, sheep, cattle, and dog)	Lin et al., 2011

A large collection of serological tests have been designed, using a wide variety of antigens. Most often, these tests were developed to detect *T. gondii* specific IgM or IgG antibodies in the sera of humans and animals. Although different formats of serological tests were designed, enzyme-linked immunosorbent assays (ELISA) and agglutination tests were the most common. In general, ELISA tests are more sensitive than agglutination tests, but they often require a species specific secondary antibody that recognize the IgM or IgG antibody of the animals to be tested. Agglutination tests are superior to ELISA in that they do not need any secondary antibodies, therefore are suitable for on-site diagnosis. Among the different ELISA formats, indirect ELISAs with native or recombinant *T. gondii* antigens to catch specific antibodies from infected hosts are the most common (Wu et al., 2009; Dai et al., 2013; Wang Z. et al., 2014). Many different antigens have been tried for this purpose, which included native antigens such as, the whole tachyzoite soluble extract and excretory-secretory antigens, as well as recombinant antigens such as, surface proteins (SAG1, SAG2, etc.), secretory proteins from different organelles (MIC3, GRA1, GRA7, etc.), and others (listed in Table 1 with corresponding references). In addition to single antigens, chimeric antigens containing epitopes from multiple proteins have also been tried and thought to be superior to single antigens (Dai et al., 2013; Wang Z. et al., 2014; Feng et al., 2016). To bypass the requirement of species specific secondary antibodies in indirect ELISAs, protein A or protein G from *Staphylococcal aureus* were used to functionally replace those secondary antibodies, due to their high binding affinities to immunoglobulins from a wide range of animals. Such ELISAs were called AG-ELISAs (Zhang et al., 2010). Besides indirect ELISAs, other formats of ELISAs, such as, double-sandwich ELISA were also developed (Lu et al., 2006). Although there was no solid and direct comparison between these different types of ELISA tests, from literature it seems like SAG and GRA7 based indirect ELISAs are the most reliable. The sensitivity and specificity were claimed to be nearly 100% and above 96%, respectively (Lu et al., 2006; Sun et al., 2015).

Like ELISAs, agglutination tests were also designed in a number of different ways (Table 1). Traditional agglutination tests (called modified agglutination tests) use fixed tachyzoites as agglutogen, which are widely used but difficult to standardize, due to the variations during tachyzoites preparation (Al-Adhami et al., 2016; Feng et al., 2016). To improve, different recombinant antigens derived from *T. gondii* were used to coat and sensitize red blood cells or latex particles, which were then used as agglutogen. Such testes are called indirect hemagglutination assays (IHA) and latex agglutination tests (LAT), respectively (Feng et al., 2016). The antigens used to sensitize agglutination particles were more or less the same as the ones used in indirect ELISAs (Chen et al., 1980; Yu, 2001; Jiang et al., 2008). In addition to agglutination tests and ELISAs, there are other serological methods reported, such as, indirect fluorescent assays (IFA) (Wang Y. B. et al., 2012). More recently, rapid diagnostic methods using immune colloidal gold technique were developed (Feng et al., 2016).

Although numerous *T. gondii* antibody detecting methods were designed in the lab, the majority of them were not subject to extensive evaluation to determine their value of clinic use. This is particularly the case for the assays to detect animal toxoplasmosis. Because of the high demand for TORCH (*Toxoplasma*, Rubella virus, Cytomegalovirus, and Herpes simplex virus) screens, China Food and Drug Administration (CFDA) issued certificates to a number of manufactures to make *Toxoplasma* specific IgM and IgG detecting kits for the diagnosis of human toxoplasmosis. However, published work assessing their sensitivity, specificity and Youden index indicated that, except for a few, the majority of these domestic kits were not of high enough quality to be useful in clinic (He et al., 2008). Especially for IgM detecting kits, the Youden index for many of them were close to zero (Jiang et al., 2003), therefore were of no value for clinic use. For the diagnosis of animal toxoplasmosis, so far there is no method certified by the China Institute of Veterinary Drug Control. Therefore, efforts are still needed to translate some of the laboratory assays to reliable and commercially available products for the diagnosis of toxoplasmosis in China. For the same reason, the sero-prevalence results discussed below only represent rough estimates of *T. gondii* infections in China.

In addition to the antibody detecting methods, there were also reports using immunological methods to detect *T. gondii* antigens in sera or other tissue extracts. For instance, a rapid modified ELISA was developed to test *T. gondii* antigens in humans, which included 5,428 sera, 548 cerebrospinal fluid and two breast milk samples. The lowest concentration of antigen detected was as low as 31.2 ng/mL without cross-reaction with antigens from other protozoa, trematodes, or nematodes (Chen R. et al., 2008). It is worth noting that detecting *T. gondii* antigens by immunological methods has limited success, due to the low levels of antigens exist and limited sensitivity of the tests.

To increase the sensitivity of pathogen detection, molecular biology methods based on nucleic acids detection were developed in recent years. Most of them were based on specific amplification of *T. gondii* DNA sequences, which include: conventional PCR, loop-mediated isothermal amplification (LAMP), nested-PCR, and real-time PCR (RT-PCR) etc. (Lin et al., 2012; Qu et al., 2013b). The target sequences for amplification often include the 529 bp repeat fragment, ITS-1, B1 gene, etc. Among these tests, LAMP is the most sensitive and does not require expensive equipments (Yang et al., 2008; Lin et al., 2012; Lai et al., 2014), therefore is suitable for on-site uses. However, it can be contaminated easily and give high rates of false positive reports.

## Sero-prevalence studies in humans

**Figure 2:**
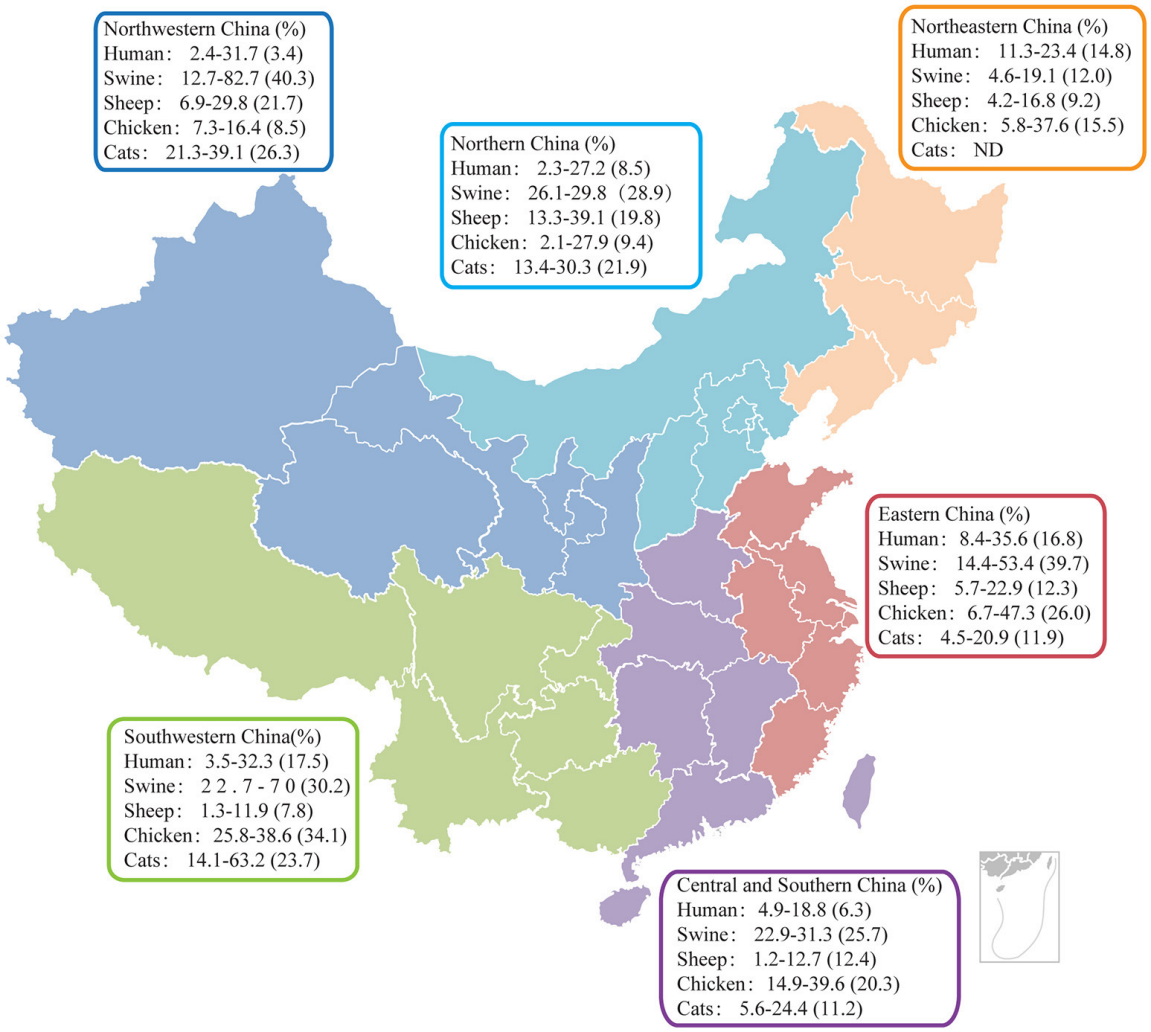
Results of representative serological studies to estimate the prevalence of *T. gondii* in humans and animals in China. Data collected from both Chinese and English publications after the year 2010 were analyzed and graphed on the map of China, which was divided into six regions for data presentation. Sero-prevalence range and average (in parentheses) for each animal in each region are shown. ND, Not determined.

As an opportunistic pathogen, *T. gondii* posts health threats to the public, particularly to individuals with reduced immune functions. China has a large susceptible population: the number of AIDS patients increased from 650,000 in 2005 to 780,000 in 2011 (Wu, 2011; Ding et al., 2017), the number of women at childbearing age was estimated to be around 375.8 million in 2013 (Ding et al., 2017). Over the last a few decades, epidemiological surveys have been frequently done to monitor the prevalence of *T. gondii* in China. The average infection rate in humans is close to 10% but it varies significantly between different geographic regions and different populations (Xiao et al., 2010; Li et al., 2011; Sun et al., 2013; Cong et al., 2015a; Li H. L. et al., 2015; Wang L. et al., 2015; Wang S. et al., 2015), etc. Some of the representative results are shown in Figure 2. It is worth noting that the sero-prevalence of human toxoplasmosis in China is lower than the world average (10 vs. 25–30%), which is likely due to the unique eating habits (boil before eating/drinking) of Chinese people (Howe and Sibley, 1995; Yu, 2000).

Years of sero-prevalence studies seem to suggest that *T. gondii* infection rates in humans increased over the last 30 years in China. For example, the overall infection rate was 5.2% in the first national survey implemented between 1988 and 1992 (Yu et al., 1994), then rose to about 8% between 2001 and 2004 (Zhou et al., 2008). Possible reasons for such change include increased meat consumption and pet cat numbers as a consequence of economy development. At present, it is estimated that 10% of humans in China are infected, but the distribution is not even. For example, due to the unique culture and eating habits, people from certain ethnic groups have significant higher infection rates than others. Thirty percent of the Bai people in Yunnan province was sero-positive, compared to 10% in Han people in the same region, which is likely due to the raw pork eating culture of the Bai people (Li H. L. et al., 2015). For the same reason, the prevalences in Miao (25.4%), Buyi (25.3%), and Mongol (17.1%) ethnic groups are also higher than other groups (Xu et al., 2005). In addition, people with certain careers are also at higher risk, herdsmen, veterinary, meat processing workers, and slaughterhouse workers tend to be more frequently infected than general public (Chen et al., 2005; Huo et al., 2007; Yu et al., 2007).

Serological tests targeting special groups of people were also frequently done, the best known of which is the TORCH examination in pregnant women. TORCH test is highly recommended by many hospitals, and is mandatory in some cities in China. It should be pointed out that the *Toxoplasma* examination part of TORCH is over simplified, only testing the presence of *T. gondii* IgM and IgG antibodies in a single test to inform the infection status. Since IgM may last more than a year, IgM positive women do not necessarily acquire the infection during pregnancy (Feng et al., 2016). As such, medical decisions made from a single IgM/IgG test are not always appropriate. Nevertheless, routine TORCH screens found that on average 0.3% of pregnant women were IgM positive, and 5.3% were IgG positive for *T. gondii* (Ding et al., 2009, 2015; Yang et al., 2014; Liu T. et al., 2017). A certain portion (although the exact number is not clear) of the IgM positive women were predicted to be infected after conception, which is high likely to lead to poor obstetric outcomes, or increase the risk congenital toxoplasmosis to newborns (Zhou et al., 2011a; Peng et al., 2015; Guan, 2017). Surveys also suggested that sero-prevalence of *T. gondii* in infertile couples is higher than that in healthy couples (Zhou et al., 2002), although the relationship between *T. gondii* infection and infertility is currently unknown. Sero-prevalence studies in hospitalized patients suggested that infection rates in AIDS patients, cancer patients, and patients in intensive care units were almost two-fold of that of health people (Xiao et al., 2010; Jiang C. et al., 2015; Zhang et al., 2015). The exact reasons for the higher sero-prevalence in such patients are currently unknown, but these results imply that patients with compromised immune functions may pick up infections and sero-convert more frequently. A recent meta-analysis on AIDS patients worldwide also showed the same trend (Liu L. et al., 2017; Liu T. et al., 2017).

## Animal toxoplasmosis

China has one of the highest biodiversities in the world, with a wide variety of landscapes and animal populations. Using mostly indirect ELISAs and agglutination tests, sero-prevalence of *T. gondii* in farm and wild animals was estimated over the years. In general, the prevalence in animals was much higher than that in humans. Representative results from epidemiological studies performed after the year 2010 were summarized in Figure 2, which provide a rough estimate for the current status of animal toxoplasmosis in China.

Among the major animals monitored, pigs were the most frequently infected by *T. gondii*. Roughly 30–50% of pigs raised in China are sero-positive for *T. gondii* (Yu et al., 2011; Wu et al., 2012; Xu Y. et al., 2014), and this number can reach 70% in some areas and farms (Li Y. N. et al., 2015). Due to the facts that China is the biggest pig-raising country (more than one billion pigs were raised and butchered each year) and pork-consuming country in the world, high *T. gondii* prevalence in pigs lead to serious economic lossess and public health concerns. First of all, As mentioned above, pigs are susceptible to *T. gondii* infection. In late 1970s and early 1980s, the “high fever with unknown reason” syndrome caused by *T. gondii* killed a large number of pigs in Yangtze River delta (Shanghai, Jiangsu, and Zhejiang) (Ren and Bao, 1982). Even nowadays, abortions caused by congenital swine toxoplasmosis are still very common, leading to huge economic losses (Fan, 2017; Wu, 2017). In addition, high *T. gondii* prevalence in pigs also increases the risks of human infection through meat consumption (Wang H. et al., 2012), since pork is the main meat Chinese people consume.

Among other meat producing animals, sheep and chickens are at the top of the list in terms of *T. gondii* infection rates. Northern and northwestern parts of China are the main regions for sheep production. Sero-prevalence in sheep is also the highest in these regions, reaching 20% (Figure 2), likely as a consequence of the widespread of *T. gondii* oocysts in the environment (Yang et al., 2017). In other regions, infection rates in sheep are about 10% on average (Wu et al., 2011a; Xu P. et al., 2014; Zou et al., 2015; Li et al., 2016; Zhang N. et al., 2016). Given the fact that among all farm animals, sheep are probably the most susceptible to *T. gondii* infection, economic losses caused by abortions and other diseases from sheep toxoplasmosis are predicted to be huge, but there is no reliable estimate so far. In addition, lamb meat is the major meat used in barbecue in China. Because of the unique processing style, barbecue often does not kill all pathogens in the meat. As such, high prevalence of *T. gondii* in sheep may make sheep an important source for human infections. Interestingly, *T. gondii* is also fairly prevalent in chickens, particularly in the south of China, over 20% of chickens are sero-positive (Zhao et al., 2012; Liu R. Z. et al., 2013). Beef is another major meat people consumed in China, but the sero-prevalence of *T. gondii* in cattle (below 10% on average) is significantly lower than that in pigs or chickens (Yu et al., 2006; Zhu et al., 2017). In addition, cattle are generally fairly resistant to *T. gondii* infection. Therefore, beef is probably not a significant source of human infections.

Cats, as definitive hosts for *T. gondii*, play a critical role in its transmission. Pet cats in particular, are important sources for human infections due to their intimate association with humans. Sero-prevalence studies, using mostly ELISAs and agglutination tests, suggested that cats were also infected with *T. gondii* with relatively high frequencies. The infection rates vary significantly, depending on the regions and cat types. In general, stray cats were much more frequently infected than pet cats (Ding et al., 2017). In one study, 43 stray cats in Shanghai examined by IHA were found to be infected at 100% (Chen, 2010). Judging from reported results, the average sero-positivity of housed cats were somewhere between 15 and 25% (Zhang et al., 2009; Wu et al., 2011c; Ding et al., 2017). But it should be pointed out that, unlike animals (pigs, chickens, etc.) that are concentrated in farms, cats are more randomly distributed and sero-prevalence studies in cats from selected regions may not reflect the overall picture. In our opinion, the sero-positivity was likely underestimated, due to the fact that most of the studies did not include samples from rural areas, where the prevalence is predicted to be higher than urban regions. There were also sporadic reports on the sero-prevalence of *T. gondii* in pet dogs. Generally speaking, infection in pet dogs is common but is not as frequent as in pet cats, and is around 10% on average (Wu et al., 2011b; Yang et al., 2013; Gao Y. M. et al., 2016).

There are also studies reporting sero-prevalence of *T. gondii* in wild animals such as, rodents, bats, and gulls etc. (Yuan et al., 2013a; Zhang Y. et al., 2013; Miao et al., 2014). However, these studies are often regional and static. Nonetheless, these sporadic studies suggested that wild animals were frequently infected. For example, sero-prevalence in rodents like *Microtus fortis* was as high as 50% in Jilin province (Zhang Y. et al., 2013), and is 29% in Hunan province (Zhang et al., 2004). Similarly, infection rates in bats and black-headed gulls are around 20% (Yuan et al., 2013a; Miao et al., 2014). These results, along with the epidemiological studies in farm animals and pets, indicated that *T. gondii* is present in the environments and food chain in China, at similar frequencies as in other parts of the world (Dos Santos et al., 2010; De Berardinis et al., 2017). However, human infection rates in China are lower than the world average, which likely ascribe to the unique eating habits of the Chinese people.

## *T. gondii* in environments

High prevalence of *T. gondii* in animals is correlated with the wide spreading of parasites in environments, most likely in the form of oocysts. Indeed, when nucleic acids based detection methods were used to assess the presence of *T. gondii* DNA, environmental samples were examined as positive at fairly high frequencies. In 2012, two studies looked at the presence of *T. gondii* DNA in soil samples collected from pig farms and public parks in central China by PCR and LAMP, and found *T. gondii* contamination in 16–23% of the samples from parks, and 21–38% of the samples from pig farms, depending the methods used (Du et al., 2012a,b). Similarly, more than 9,000 soil samples collected from northeastern China were analyzed by real-time quantitative PCR and found over 30% of them to be positive (Gao X. et al., 2016). Presumably the detected *T. gondii* specific DNA was derived from oocysts, however it remains unknown how often viable oocysts can be isolated from such environmental samples. Given the environmental resistance of oocysts, a significant portion of the DNA positive samples were likely to contain viable oocysts, which may explain the high infection rates in animals. On the other hand, the PCR based methods to detect oocysts in environments are very sensitive to contaminations, extra caution needs to be taken in interpreting such data. Alternative methods such as, oocyst enrichment and isolation are necessary to estimate the spread of oocysts in environmental samples.

## Genotypes of *T. gondii* strains circulating in China

Numerous efforts were taken to understand the genotypes and population structures of the *T. gondii* strains prevalent in China. Generally, two methods were used for this purpose. Either isolated live strains and genotyped them, or extracted DNA from infected tissues and then used the DNA samples for genotyping, with the latter being much more common. Either way, the isolates were often genotyped by PCR-RFLP based on 11 genetic markers (SAG1, 5′-SAG2 and 3′-SAG2, alternative SAG2, SAG3, BTUB, GRA6, c22-8, c29-2, L358, PK1, and an apicoplast locus Apico; Su et al., 2006). Consistent with the large territory and great biodiversities of hosts in China, many different genotypes were discovered, which were summarized in Table 2. It is obvious that the dominating genotype in China is ToxoDB #9 (also called Chinese I or China I), moren than half of the isolates belong to this genotype (Li Y. N. et al., 2015). ToxoDB #9 is the dominating strain type in almost all examined hosts (Table 2). The next common one is ToxoDB #10 (Type I), which is the classic type 1 genotype (Zhou et al., 2009; Wang et al., 2013). However, it should be pointed out that most studies only reported the detection of DNA corresponding to ToxoDB #10 strains, only a few live type I parasites (TgHuZS2 and TgCtxz1) were isolated and preserved (Wang et al., 2013). Therefore, the actual frequency of such strains in China remain to be determined. ToxoDB #9 and #10 strains probably account for 90% of the strains examined in China. This situation is similar to North America and Europe in that a few genotypes dominated the population (Minot et al., 2012). However, unlike the clonal nature of the type I, II, and III strains in north America and Europe, there are probably multiple subtypes in the ToxoDB #9 group, since different ToxoDB #9 isolates have quite different properties (such as, acute virulence in mice). Using multilocus sequence typing, a most recent study suggested that ToxoDB #9 isolates could be divided into at least two (potentially more) groups: Chinese I and Chinese III, with Chinese III being more closely related to classic type 1 strains (Gao et al., 2017). In addition, the dominating ToxoDB #9 strains seem to be distinct from the strains isolated from other parts of the world. Using three independent sets of polymorphic DNA markers to estimate the phylogenetic distances among 950 isolates collected from all over the world, the TgCtPRC04 strain representing ToxoDB #9 isolated from China was separated from other strains and formed a unique haplogroup (Su et al., 2012). Whole genome sequencing was done on another ToxoDB #9 strain TgCtPRC2 (which was isolated from a cat and also belonged to the same haplogroup as TgCtPRC04) and the data of which was deposited in ToxoDB, which facilitated the biological studies of ToxoDB #9 strains.

**Table 2:** Genotypes of *T. gondii* parasites prevalent in China.

**Host**	**Tissue origin**	**Type (Number)**	**Frequency**	**References**
Cancer patients	Diseased tissues	ToxoDB #9 (8) ToxoDB #10 (9)	ToxoDB #9: 65% ToxoDB #10: 35%	Cong et al., 2015b
	Serum samples	ToxoDB #9 (9)		Wang L. et al., 2015
Pigs	Hilar lymph nodes	ToxoDB #10 (5 ToxoDB #3 (1) ToxoDB #9 (21)	ToxoDB #9: 83% ToxoDB #10: 11% ToxoDB #3: 4% Others: 2%	Zhou et al., 2010; Zhou et al., 2011b; Jiang H. H. et al., 2013; Jiang W. et al., 2013; Jiang et al., 2016
	Retail meat	ToxoDB #9 (1) ToxoDB #213 (1)		Wang H. et al., 2012
	Blood, heart and brain	ToxoDB #9 (3) ToxoDB #3 (1) ToxoDB #9 (13)		Li Y. N. et al., 2015; Wang D. et al., 2016
Cats	Brain, tongue, Heart, liver, Blood, feces	ToxoDB #9 (29) ToxoDB #225 (2) ToxoDB #1 (4) ToxoDB #2 (1) ToxoDB #3 (1) ToxoDB #20 (1) ToxoDB #17 (1)	ToxoDB #9: 74% ToxoDB #1: 10% ToxoDB #225: 5% Others: 11%	Qian et al., 2012; Liu R. Z. et al., 2013; Liu T. et al., 2013; Wang et al., 2013; Li et al., 2014; Tian et al., 2014; Li Y. N. et al., 2015; Yang et al., 2015; Cong et al., 2016a
**RODENTS**				
*Microtus fortis*	Lung	ToxoDB #10 (2) ToxoDB #9 (2)	ToxoDB #9: 53% ToxoDB #10: 35% Others: 12%	Zhang et al., 2014
Qinghai vole Plateau pika Tibetan ground-tit	Brain	ToxoDB #10 (4) New genotype (2)		Zhang X. X. et al., 2013
Rats and mice	Brain	ToxoDB#9 (7)		Yan et al., 2014
**OTHERS**				
Wild birds	Breast, muscle	ToxoDB #10 (1) ToxoDB #1 (2)	ToxoDB #9: 44% ToxoDB #10: 37% ToxoDB #3: 7% ToxoDB #2: 5% Others: 7%	Huang et al., 2012
Pet birds	Brain	ToxoDB #3 (1)		Cong et al., 2014
	heart	ToxoDB#9 (1) ToxoDB#2 (1) ToxoDB#10 (1)		Chen et al., 2015
House sparrows	Heart, brain, lung	ToxoDB #3 (1) New genotype (1)		Cong et al., 2013
Domestic rabbit	Brain, spleen, liver	ToxoDB #2 (1)		Zhou et al., 2013
Bats	Lung, heart, liver, spleen, stomach, intestine or kidney	ToxoDB #10 (4) ToxoDB #9 (1)		Jiang et al., 2014
	liver	ToxoDB#10 (3) ToxoDB#9 (5)		Qin et al., 2014
Black goats	Liver, lung, lymph nodes	ToxoDB#10 (7) ToxoDB#9 (1)		Miao et al., 2015
Sika deer	Liver, lung, muscle	ToxoDB#9 (6)		Cong et al., 2016b
Farmed minks	Brain	ToxoDB#9 (5) ToxoDB#3 (1)		Zheng et al., 2016

## Vaccine developments

Starting in 1990s, with the rapid progress of molecular biology techniques, a significant amount of *T. gondii* research work was on vaccine developments, particularly subunit vaccines. Some of the representative studies were summarized in Table 3, which shows that there are mainly three types of vaccines tried: recombinant protein vaccines, DNA vaccines, and live vaccines. The core antigens used for recombinant protein vaccines and DNA vaccines are very similar, mostly surface proteins (SAGs) and proteins from secretory organelles such as, dense granules (GRAs), rhoptries (ROPs), and micronemes (MICs) (see Table 3 for examples and references). These two types of vaccines only differ in the way the core antigens being delivered: for recombinant protein vaccines, the core antigens were administrated in the form of purified proteins, often along with chemical adjuvant to boost the immune response; DNA vaccines were administrated in the form of DNA (plasmids or viral vectors) that expressed the core antigens in hosts, sometimes using cytokine (IL-12, IL-15, etc.) expressing constructs as adjuvants (Zhang et al., 2007; Cui et al., 2008; Chen et al., 2014a). Most of these vaccines elicited Th1 immune responses and had some degree of protections against parasite infections, increasing survival rates, and/or time or reducing cyst burdens (Yuan et al., 2011b; Zheng et al., 2017). Nonetheless, none of these subunit vaccines in the current form offered enough protection to be used in clinic.

**Table 3:** *T. gondii* vaccine candidates designed by scientists in China.

	**Antigens/parasite strains**	**Effect**	**References**
DNA and vector vaccines	SAG1	Increased survival time to 20.38 ± 3.38 days vs. control (13.25 ± 1.16 days)	Chen et al., 1999
	SAG1, SAG2 linked to A2/B subunits of cholera toxin	40% survival rate in immunized mice	Cong et al., 2005
	SAG1, ROP2 with IL-12 as adjuvant	Increased survival time to 22 days vs. control (4–8 days)	Zhang et al., 2007
	SAG1-ROP2-SAG2 co-deliveried with IL-12	Increased survival rate	Cui et al., 2008
	SAG1, GRA1, GRA2, GRA4 antigen segments	The survival rate of BALB/c and C57BL/6 mice were 100 and 40%, respectively	Liu et al., 2009
	SAG2C, SAG2D, SAG2X	Reduction of cyst burden (77%) in the brain	Zhang M. et al., 2013
	MIC3 (suicidal vector pSCA1)	Increased the survival time to 15 days vs. control mice (5 days)	Fang et al., 2009
	MIC3 (recombinant pseudorabies virus rPRV)	The survival rates of BALB/c mice injected with rPRV-MIC3 alone was 50%	Nie et al., 2011
	MIC3, SAG1 (baculovirus vaccine BV-MIC3+BV-SAG1)	50% of the mice survived	Fang et al., 2012
	MIC6	Increased survival time to 13.3 ± 1.2 days vs. control mice (7 days)	Peng et al., 2009
	MIC8	Increased survival time to 10.3 ± 0.9 days vs. control mice (5 days)	Liu M. M. et al., 2010; Liu Y. et al., 2010
	MIC11	Increased the survival time to 15 days vs. control mice (8–10 days)	Tao et al., 2013
	MIC13	Increased survival time to 21.3 ± 11.3 days vs. control mice (5–10 days) and reduced number of cysts in brain of mice (57.14%)	Yuan et al., 2013b
	ROP9	Increased survival time to 12.9 ± 2.9 days vs. control mice (6 days)	Chen et al., 2014b
	ROP16	Increased survival time to 21.6 ± 9.9 days vs. control mice (7 days)	Yuan et al., 2011a
	ROP18	Increased survival time to 27.9 ± 15.1 days vs. control mice (7 days)	Yuan et al., 2011b
	ROM1	Increased survival time to 12.5 ± 0.7 days vs. control mice (5 days)	Li et al., 2012
	GRA6 with levamisole as adjuvant	53.3% survival	Sun et al., 2011
	eIF4A	Increased survival time to 23.0 ± 5.5 days compared to control mice (7 days)	Chen et al., 2013
	MIC3, GRA1	Increased survival time to 12–19 days (15.7 ± 1.88) vs. control group survived for 3–5 days (4.5 ± 0.38)	Gong et al., 2016
	MIC3, ROP18	Increased survival time to 14–19 days vs. control mice (7 days)	Qu et al., 2013a
	Aspartic protease 1	Increased the survival time to 16 days vs. control (7 days)	Zhao et al., 2013
	CDPK1 with a plasmid encoding IL-15 and IL-21	Increased survival time to 19.2 ± 5.1 days vs. control (6 days) and reduced the number of brain cysts (72.7%)	Chen et al., 2014a
	NTPase-II (pDREP)	71.4% reduction in brain cysts	Zheng et al., 2017
Recombinant protein vaccines	ROP5	Increased survival time to 12.1 ± 3.4 days vs. control (6 days)	Zheng et al., 2013
	ROP17	Lower liver and brain parasite burdens (59.17 and 49.08%, respectively) and increased survival time by 50%	Wang H. L. et al., 2014
	ROP18, ROP38 encapsulated in poly (lactide-co-glycolide)	81.3% reduction of tissue cysts	Xu et al., 2015
Live vaccines	*T. gondii* temperature-sensitive mutant	Induced protective immunity in an ocular toxoplasmosis model	Lu et al., 2009
	*Eimeria tenalla* sporozoites expressing TgSAG1	Induced Th1 immune response and prolonged survival of mice	Tang et al., 2016

Live vaccines were thought to be the most effective against *T. gondii*, with the good example of “Toxovax,” although this vaccine is also not perfect (Buxton and Innes, 1995; Burrells et al., 2015). Design of *T. gondii* vaccines using live parasites were also tried in China, but only with limited success. For example, A temperature-sensitive mutant of RH (ts-4) was tested as a live vaccine for ocular toxoplasmosis in a mouse model. After intracameral inoculation, this strain did provide protective immunity against wild type parasite infection (Lu et al., 2009). However, the strain by itself also caused ocular pathology, preventing its use as a vaccine. Live vaccines were also designed using other parasite species as antigen delivery vectors. For example, transgenic *Eimeria tenella* expressing TgSAG1 was tested as vaccine to protect *T. gondii* infection in chickens and mice. Although it did elicit a Th1-dominant immune response that restricted *T. gondii* proliferation, the protective immunity it offered was limited (Tang et al., 2016). More recently, with the wide use of CRISPR/CAS9 based genome editing system, generating gene deletion mutants to be used as live vaccines became popular, which is also a feasible approach given the success of uracil auxotrophs (Fox and Bzik, 2002).

## Molecular basis for pathogenesis

Before the year 2000, the majority of *T. gondii* research in China is about epidemiology and vaccine design, very little was done toward the understanding of parasite biology or mechanisms of pathogenesis. After entering the new century, basic research became more active. By looking at the papers Chinese scientists published during the last 15 years, it is obvious that the vast majority of the limited work was about host-parasite interactions, or what influence parasites had on host and host cells. Quite a few transcriptomic and proteomic studies examined the gene expression and protein abundance changes of the hosts upon parasite infection. For examples, RNA-Seq was used to look at the gene expression changes in cat liver, mouse liver and pig peripheral blood mononuclear cells (PBMCs) after infection (He et al., 2016a; Zhou et al., 2016a,b). Similarly, iTRAQ-based proteomic analysis on mice liver suggested that the relative abundances of hundreds of proteins were changed upon parasite infection, many of which were involved in key metabolic pathways (He et al., 2016b). These omics studies did generate a large amount of data that were informative, but follow-up studies are required to figure out the biological significances of these data. More recently, with the development of genome editing techniques, increasing amount of work started to address the biological properties of the parasite. Using the CRISPR/Cas9 technology, leucine aminopeptidase, calcium-dependent protein kinases, and rhoptry proteins were inactivated to check the functions of these genes during parasite growth and development (Zheng et al., 2015; Wang J. L. et al., 2016, 2017). Today, studies in China cover most fields of *T. gondii* research. In this review, we only focused on the studies of ToxoDB #9 strains, which are almost specific to China.

As mentioned above, strains isolated from China are dominated by the ToxoDB #9 genotype. However, strains belonging to this genotype may display different properties. TgCtwh3 and TgCtwh6 are probably the best characterized ToxoDB #9 strains, and they show different virulence in mice, with TgCtwh3 being significantly more virulent than TgCtwh6 (Wang et al., 2013). In addition, TgCtwh3 was shown to induce alternative activation of mouse macrophages with STAT6 phosphorylation, whereas the TgCtwh6 elicited classical activation of mouse macrophages with nuclear translocation of NF-κB (Zhang A. M. et al., 2013). In order to understand why the two strains with the same genotype had these differences, both strains were subject to whole genome sequencing and compared to that of type 1 strain GT1. Although single nucleotide polymorphisms (SNPs) and insertions/deletions (indels) were found in hundreds of genes in TgCtwh3 and TgCtwh6 strains, these two strains were genetically more similar to each other than to GT1. GRA3 and RON3 were shown to have drastic expression differences between the two strains, but whether or not they contribute to the phenotypic difference is still unknown (Cheng et al., 2015). A recent study using multilocus sequence typing suggested that ToxoDB #9 strains could be divided into multiple sub-groups. However, using this typing method, TgCtwh3 and TgCtwh6 were still grouped together, although they display different phenotypes. As the authors of this work implied, perhaps genotyping using neutral genetic markers is not very useful in predicting pathogenic phenotypes (Gao et al., 2017). Therefore, further studies are required to dissect the underlying molecular mechanisms for such phenotypic differences, as a way to better understand the biology of ToxoDB #9 strains.

## Chinese herbal medicines for *T. gondii* intervention

For decades, inhibitors of folic acid metabolism such as, pyrimethamine and sulfonamide were the primary drugs to treat acute toxoplasmosis. But due to strong side effects and other limitations, these drugs are far from ideal. Developing new anti-*Toxoplasma* interventions is a long lasting task in the field and scientists in China contributed to this effort. Noticeable achievements include the studies of Chinese herbal medicine against *T. gondii* (Table 4). As early as 1990, the effect of artemisinin and its derivatives on *T. gondii* growth was tested *in vitro*, and found that at 0.4 μg/ml artemisinin blocked plaque formation on host cell monolayers (Ke et al., 1990). Follow-up work was done to test the effect of artemisinin in controlling toxoplasmosis *in vivo* in animal models (Shen et al., 2003; Yang and Wan, 2006). The results were not exactly the same from different studies but all seemed to show that artemisinin was not as effective as expected, and some derivatives were more potent than the others (Dunay et al., 2009).

**Table 4:** Effects of herbal medicines or their active components on *Toxoplasma gondii*.

**Active ingredients**	**Dosage**	**Effect**	**References**
Artemisinin	0.4 μg/mL (5 days) 1.3 μg/mL (14 days)	Inhibit plaque formation *in vitro* Eliminate all parasites *in vitro*	Ke et al., 1990
Ginkgolic acids	167.1 μg/ml	No visible parasites in HFF cells after 48 h of exposure to ginkgolic acids (isolated from the *Ginkgo biloba* sarcotesta)	Chen R. et al., 2008
Inontus obliquus polysaccharide	3 mg/10 g/d	Decreased testicular spermatogenic cell pathology damage caused by *Toxoplasma* infection	Liu Y. et al., 2010
Radix glycyrrhizae	5 g/Kg	While combined with sulfachloropyrazine-sodium (SPZ), the survival rate of mice was up to 50%	Jiang W. et al., 2013
Oxymatrine, matrine	100 mg/Kg	Decreased the number of tachyzoite (45.2 and 53.8%) and increased survival rate of mice to 67%	Zhang X. et al., 2016

In addition to artemisinin, which was isolated from *Artemisia annua*, extracts from many other herbs such as, *Glycyrrhiza, Scutellaria*, and *Ginkgo biloba* etc, were also reported to have anti-*Toxoplasma* effects (Chen S. X. et al., 2008; Gong et al., 2011). However, in most cases, the active compounds in these extracts were not known and deserve further research. There are a few occasions where the active compounds were reported. For example, oxymatrine (OM) and matrine (ME), the main alkaloids in the *Sophora leguminous* plants, were shown to restrict *T. gondii* growth both *in vivo* and *in vitro*: both OM and ME showed high anti-*T. gondii* activity and low toxicity to Hela cells than spiramycin (SPY); treating infected mice with oxymatrine or matrine increased the survival rates, although not to 100% (Zhang X. et al., 2016). This shows the potential of using natural products to treat toxoplasmosis, but obviously we need to dig further to find out and optimize the active components.

## Conclusions and perspective

Since the first isolation and discovery of *T. gondii* in China in 1955, Chinese scientists have been working on this parasite for over 60 years. Tremendous amount of work has been done on the development of diagnostic strategies, subunit vaccines, and epidemiology surveys. It is clear that *T. gondii* is widespread in China. In particular, pigs and chickens, the main meat source for Chinese citizens, as well as cats, a popular pet people keep, are among the most frequently infected animals, which greatly increase the chance of human infections. But thanks to the unique eating/cooking habits, human infection rates in China are below the world average. A large number of vaccine candidates in different forms were designed, but frankly speaking, none of them is good enough to be used as a vaccine in the current form. Years of research in the vaccine design field seems to suggest that live attenuated parasites may be the best way to achieve good *T. gondii* vaccines. With the improvements of genetic modification techniques in *T. gondii*, work on designing live attenuated vaccines becomes more common, significant progress in this direction is expected.

In terms of diagnostics, although lots of efforts have been taken and a variety of assays were developed, very few of them were translated into standardized commercial products or technical standards. As a consequence, many veterinary clinics use home-made protocols for *T. gondii* diagnosis. For the toxoplasmosis examination in humans during pregnancy, well-established reference laboratories are still lacking in China. Most hospitals use a single IgM-IgG test to judge the risk, which is not reliable and may lead to false positive results and unnecessary termination of pregnancy. In addition, diagnostic products, especially IgM detecting kits, from domestic manufacturers are not yet ideal for clinic uses. Therefore, efforts are needed to translate the laboratory assays to standardized products for accurate and consistent diagnosis. Reference laboratories should be established to help local clinics and hospitals to get reliable test results for human samples. In addition to these, new diagnostic strategies, which can not only tell the infection status but also inform the source of infection (oocysts vs. tachyzoites/bradyzoites; Santana et al., 2015), may be needed for better risk factor association analysis.

On the basic research side, although so far the contribution from Chinese scientists to the understanding of biology, as well as mechanisms of pathogenesis of *T. gondii* is limited, it is obvious that the research activities are really booming after the year 2000. Currently, there are groups working on the host mechanisms that restrict parasite infections (Qin et al., 2017), parasite mechanisms of immune evasion (Xue et al., 2017), molecular basis for *T. gondii* invasion (Wang Y. et al., 2014; Wang M. et al., 2017), growth and differentiation (Shen et al., 2016), etc. Besides these common research interests shared by the whole community, studies addressing the population structure of strains prevalent in China, the unique biological properties of ToxoDB #9 strains, and interplays between local prevalent strains and animal hosts are also required. It is expected that, after some years, breakthroughs in related fields will be made by our Chinese colleagues.

## Author contributions

MP, CL, JZ, and BS reviewed the literature and wrote the paper.

### Conflict of interest statement

The authors declare that the research was conducted in the absence of any commercial or financial relationships that could be construed as a potential conflict of interest.
